# Successful Management of Complicated Burst Abdomen With Open Abdomen Using Only Simple Saline Dressing

**DOI:** 10.1155/cris/6862550

**Published:** 2025-03-25

**Authors:** Dionizi Muganga, Francis Basimbe, Irene Nayiga, Amanda Ategeka, Paddy Malinga, Twaha Muwanga

**Affiliations:** ^1^Entebbe Regional Referral Hospital, Mother Kevin Post Graduate Medical School, Uganda Martyrs University, Nkozi, Uganda; ^2^Mother Kevin Post Graduate Medical School, St. Francis Hospital Nsambya, Uganda Martyrs University, Nkozi, Uganda; ^3^Department of Surgery, Entebbe Regional Referral Hospital, Entebbe, Uganda; ^4^Mother Kevin Post Graduate Medical School, Uganda Martyrs University, Nkozi, Uganda

## Abstract

**Introduction**: Necrosis of the rectus or lateral abdominal wall investing fascia may be associated with invasive infections or closure under extreme tension. This can lead to fascial dehiscence and evisceration of the intra-abdominal contents. Globally, abdominal wound dehiscence varies from 0.4% to 3.5% with associated mortalities reaching up to 45% in the perioperative period. Redo surgical operations and infectious complications are the major risk factors for abdominal wound dehiscence, but also presence of low albumin, glucocorticoid use, chest infections, and emergency surgeries have been also implicated. Open abdomen has been employed in incidences of trauma where a second look operation may be necessary, loss of abdominal wall, sepsis after penetrating abdominal trauma, and in cases of severe secondary peritonitis and acute pancreatitis. Patients with open abdomen are at a risk of fistula formation, sepsis, and loss of abdominal domain due to lateral fascial retraction. To reduce the mentioned complications mesh and nonmediated techniques to bridge fascia defects have been recommended with particular emphasis on biologic meshes with or without negative pressure wound therapy, component separation, or planned ventral hernia.

**Methods:** We report a case of necrosis of the rectus and abdominal wound dehiscence and its management in a sub-Saharan setting, highlighting the challenges encountered and lessons learned.

**Conclusion:** Retention sutures should be used cautiously in the management of wound dehiscence as it increases the risk of fascial necrosis in cases of intra-abdominal hypertension, as seen in our patient. In the absence of a VAC dressing, the utilization of routine saline gauze dressing promotes epithelialization over the exposed bowel and is a viable alternative to temporary abdominal closure modes of managing an open abdomen in a resource-limited setting.

## 1. Introduction

Necrosis of the rectus or lateral abdominal wall investing fascia may be associated with invasive infections or closure under extreme tension. This can lead to fascial dehiscence and evisceration of the intra-abdominal contents. Globally, abdominal wound dehiscence varies from 0.4% to 3.5% with associated mortalities reaching upto 45% in the perioperative period [1]. Redo surgical operations and infectious complications are the major risk factors for abdominal wound dehiscence, but also presence of low albumin, glucocorticoid use, chest infections, and emergency surgeries have been also implicated [2, 3]. The open abdomen has been employed in incidences of trauma where a second look operation may be necessary, loss of abdominal wall, sepsis after penetrating abdominal trauma, and in cases of severe secondary peritonitis and acute pancreatitis [4]. Patients with open abdomen are at a risk of fistula formation, sepsis, and loss of abdominal domain due to lateral fascial retraction [5]. To reduce the mentioned complications mesh and nonmediated techniques to bridge fascia defects have been recommended with particular emphasis on biologic meshes with or without negative pressure wound therapy, component separation, or planned ventral hernia [6].

## 2. Case Presentation

We present a 78-year-old female who was referred to our Regional Referral Hospital with features suggestive of intestinal obstruction. She presented with progressive abdominal distension, colicky abdominal pain, absolute constipation, and bilious vomiting that had lasted for 5 days. She reported caesarian sections surgery 40 years prior to admission. Physical examination revealed a sick elderly woman with features of dehydration however no pallor of mucosa membranes or Jaundice with a performance status of 1 on the ECOG performance status. Abdominal examination findings were of a moderately distended abdomen, a subumbilical midline scar, moving with respiration, and moderate tenderness globally with metallic bowel sounds. Digital rectal examination findings were normal. A plain abdominal X-ray showed multiple air fluid levels with distended small bowel loops, complete blood count findings were normal (WBC—6800/µl, neutrophils 65.6%, HB—12.8 g/dl, platelets 215,000/µl), urea 2.6 mmol/l, and creatinine 79 µmol/l. A diagnosis of small bowel obstruction was made and the patient was consented for laparotomy. On operation, the jejunum and ileum were clustered inform of a cocoon and adhered to lower anterior abdominal. Adhesionlysis was done with difficulty sustaining three iatrogenic perforations on the ileum. We resected about 40 cm of ileum encompassing the three perforations and an ileo—ileal end-to-end anastomosis was performed with primary abdominal wall closure.

**Figure 1:**
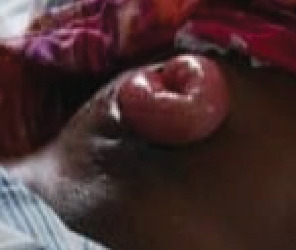
Burst abdomen postoperative Day 7.

**Figure 2:**
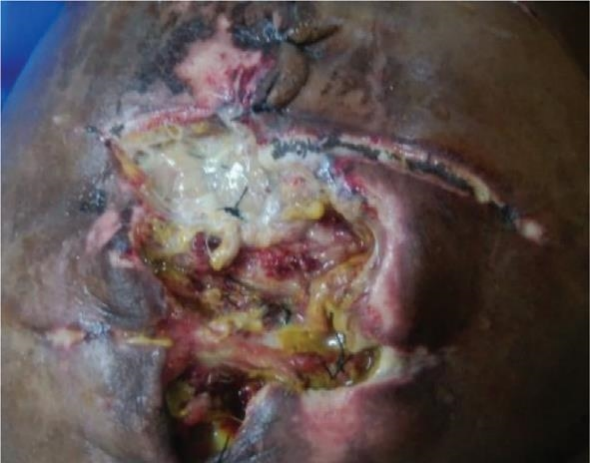
Fascial necrosis Day 3 after placement of retention sutures.

**Figure 3:**
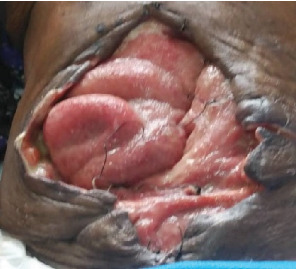
Open abdomen Day 14.

**Figure 4:**
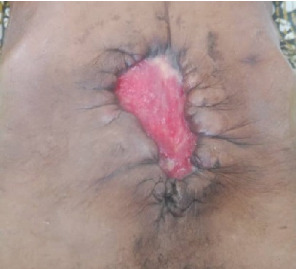
Granulating wound at hospital discharge Day 64.

**Figure 5:**
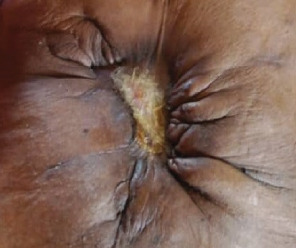
Complete healing Day 129.

The patient had good postoperative progress until postoperative Day 7 when she developed colicky abdominal pain, vomiting, and abdominal distension with wound dehiscence. A redo surgery with retention suturing was done. Day 3 after retention suturing, the patient developed generalized oedema, abdominal distension, obstipation, bilious vomiting, and ischemic necrosis of the anterior abdominal wall with sepsis, wound dehiscence, and evisceration of bowel. Laboratory evaluation revealed a low albumin of 18.5 g/dl. All retention sutures were removed, Nasogastric tube decompression and abdominal wall debridement were done creating an open abdomen. The patient received 200 mls infusions of 20% albumin in 24 h, daily saline gauze dressing over the eviscerated bowel. Granulation tissue formed over the exposed bowel. Mesh closure of the fascial defect was attempted but failed due to sepsis which in hindsight was a mistake. Thus, we continued dressing with Actisorb and iodinated gauze. Patient was discharged from the hospital after 64 days in hospital on and declared healed 129 days after the initial surgery with a residual ventral hernia.  Figure 1 shows burst abdomen postoperative day 7.  Figure 2 shows fascial necrosis day 3 after placement of retention sutures.  Figure 3 shows open abdomen day 14.  Figure 4 shows granulating wound at hospital discharge day 64.  Figure 5 shows complete healing day 129.

## 3. Discussion

Fascial dehiscence complicates the postoperative course of a significant number of patients leading to increased hospital costs, length of hospital stay, redo surgeries, and other complications [7, 8]. This patient had to undergo a redo surgery and her total length of hospital stay was 64 days

The risk factors for bowel evisceration and facial dehiscence have been widely studied and these include age greater than 65 years, surgical site infections, intra-abdominal hypertension, and emergency surgery [9]. The above risk factors were present in this patient; however, after the redo surgery with retention sutures, the patient developed paralytic ileus which led increased intra-abdominal pressures and its known that retention sutures reduce abdominal compliance thus ischemic necrosis of the abdominal wall leading fascial dehiscence [10].

Nontrauma application of open abdomen has been on the steady rise and it has been successfully utilized in the conditions of increased intra-abdominal pressure, severe intra-abdominal infection, sepsis, peritonitis, and severe acute pancreatitis. The utilization of open abdomen also poses a number of complications like fistula formation, infections, abscesses fascial retraction, dehydration, and ventral hernias. Under certain circumstances delayed primary closure may not be achieved in an open abdomen and a bridged synthetic or biologic material may be used to bridge the gap followed by skin grafting after granulation tissue formation [11]. A nonabsorbable polypropylene mesh was applied in this patient to bridge the fascial defect but failed due to infection and was removed. In this case, VAC dressing was not ideal due to exposed bowel.

In our setting, open abdomen can close secondary as long as there is adequate control of infections and other factors that impair wound healing like low albumin levels and anemia. However, this has been known to increase the length of hospital stay.

## 4. Conclusion

Fascial dehiscence still remains a nightmare for the gastrointestinal surgeon and carries a high morbidity and mortality. Retention sutures should be used cautiously in the management of wound dehiscence as it increases the risk of fascial necrosis in cases of intra-abdominal hypertension. The utilization of routine saline gauze dressing promotes epithelialization over the exposed bowel and a viable alternative to temporary abdominal closure modes for managing an open abdomen.

## Data Availability

The data underlining this study are available at the Entebbe Regional Referral Hospital and St. Francis Hospital Nsambya at the Records Department.
